# Microenvironment-associated gene HSD11B1 may serve as a prognostic biomarker in clear cell renal cell carcinoma: a study based on TCGA, RT‑qPCR, Western blotting, and immunohistochemistry

**DOI:** 10.1080/21655979.2021.1994908

**Published:** 2021-11-30

**Authors:** Di Han, Zhongjie Yu, Hong Zhang, Haipeng Liu, Bin Wang, Donmeng Qian

**Affiliations:** a Department of Pathogenic Biology, School of Basic Medicine, Qingdao University, Qingdao, Shandong, P. R. China; b Department of Special Medicine, School of Basic Medicine, Qingdao University, Qingdao, Shandong, P. R. China; c School of Public Health, Qingdao University, Qingdao, Shandong, P. R. China; d Oral Research Center, Qingdao Municipal Hospital, Qingdao, Shandong, P. R. China

**Keywords:** Immunoglobulin hydroxysteroid 11-beta dehydrogenase-1, clear cell renal cell carcinoma, immune infiltration, prognosis, tumor microenvironment

## Abstract

Clear cell renal cell carcinoma (ccRCC) is one of the most common malignant tumors worldwide. The clinical treatment of ccRCC is strongly associated with the tumor microenvironment (TME). Identifying potential markers of ccRCC is important to improve prognosis. Therefore, in the present study, the levels of immune/stromal components and the proportion of tumor-infiltrating immune cells (TIICs) were determined in 611 ccRCC samples using the ESTIMATE and CIBERSORT analytical tools. Subsequently, hydroxysteroid 11-beta dehydrogenase-1 (HSD11B1) was identified by univariate Cox regression analysis, protein-protein interaction (PPI) networks and clinical survival analysis to be associated with ccRCC prognosis. At the same time, the abundance of HSD11B1 increased significantly in ccRCC was verified by western blotting, RT‑qPCR and immunostaining analysis. Furthermore, Gene Set Enrichment Analysis (GSEA) and TME suggested that HSD11B1 was involved in TME immune-related status. Taken together, the results of the present study demonstrated that HSD11B1 is a potential prognostic biomarker associated with immune cell infiltration in ccRCC.

## Introduction

Clear cell renal cell carcinoma (ccRCC) is the third most common type of cancer of the urinary system with a high mortality rate [1,2], accounting for 2–3% of all adult cancer cases and 70% of all renal cancer cases [3–5]. Currently, surgical resection, radiotherapy and chemotherapy are the main treatment approaches to ccRCC. However, these treatment strategies do not significantly prolong the survival time of patients [6,7]. Several studies have suggested that changes of specific genes expression are highly related to the tumorigenesis and development of ccRCC [8]. Hence, investigating key gene correlated with ccRCC prognosis and identifying optimal immune-related biomarkers is crucial [9].

The tumor microenvironment (TME) is extremely complex, and consists of immune cells, various types of stromal cells, alongside with tumor cells [10–12]. Previous studies have also suggested that immune and stromal cells are the main components of TME, which are closely related to tumor progression and clinical prognosis [13,14]. Several clinical and genomic studies have also reported that ccRCC is a highly immune infiltration tumor [15]. The components of TME have an significant influence on the occurrence and progression of ccRCC [16,17]. Emerging evidence has indicated that the tumor-infiltrating immune cells (TIICs) within TME may affect therapeutic efficacy [18].

The Cancer Genome Atlas (TCGA) data of patients with ccRCC were downloaded, and the proportions of immune/stromal cells and TIICs in samples were calculated using the ESTIMATE [19] (Estimation of Stromal and Immune cells in Malignant tumor tissues using Expression data) and CIBERSORT (A general computational method for estimating the composition of TIICs populations from gene expression data) algorithms [20]. Eventually, a predictive biomarker, hydroxysteroid 11-beta dehydrogenase-1 (HSD11B1) was selected. The HSD11B1 gene encodes the type 1 isoform of 11-β-hydroxysteroid dehydrogenase, which converts glucocorticoids into the active form, which plays an important role in the regulation of cell proliferation, differentiation and immune response [21,22]. Proliferative phenotypes induced by high expression of HSD11B1 are associated with poor cancer prognosis [23]. Previous studies suggested that increased HSD11B1 expression was interconnected with poor survival of ccRCC patients, supporting that HSD11B1 have the potential to be a prognostic biomarker in patients with ccRCC receiving immunotherapy.

Our work aims to further evaluate significance of HSD11B1 expression in ccRCC prognosis and the correlation with clinicopathological characteristics using TCGA data and in vitro experiments, thereby providing additional evidence of HSD11B1 as a prognostic biomarker associated with immune cell infiltration in ccRCC.

## Material and methods

### Data collection

Transcriptome single-cell RNA sequencing profiling data (611 cases: 539 tumor cases and 72 normal cases, workflow type: HTseq-FPKM) and related clinicopathological characteristics (Table 1), including age, gender, pathological stage, grade, T/M/N classification of ccRCC samples were downloaded from TCGA database [24] (https://portal.gdc.cancer.gov/). The ESTIMATE [19] algorithm, an open-source web tool, was used to calculate the proportions of immune/stromal cells and scores in ccRCC samples using ‘estimate’ package (HTTP: // R-forge.R-project.org;Repos = Rforge, dependencies = TRUE). The scores were positively correlated with the proportion of the immune/ stromal/ ESTIMATE components in TME.

**Table 1.** Clinicopathological characteristics statistics in ccRCC patients from TCGA

Clinical characteristics		Total(537)	%
Age at diagnosis (y)	young age (≤65)	352	65.5
	old age (>60)	185	34.5
Gender	Male	346	64.4
	Female	191	35.6
Stage	I	269	50.1
	II	57	10.6
	III	125	23.2
	IV	83	15.5
	Unknown	3	0.6
T classification	T1	275	51.2
	T2	69	12.9
	T3	182	33.9
	T4	11	2.0
M classification	M0	426	79.3
	M1	79	14.7
	Unknown	32	6.0
N classification	N0	240	44.7
	N1	17	3.2
	Unknown	32	52.1
Grade	G1	14	2.6
	G2	230	42.8
	G3	207	38.6
	G4	78	14.5
	Unknown	8	1.5

### Identification of differentially expressed genes (DEGs)

Subsequently, the ccRCC samples were divided into high/low-score groups according to the median score. R (version 4.0.3) and R language packages ‘limma Bioconductor’ [25] were applied for DEGs between the high/low-score group. FDR<0.05 and |logFC|>1 were considered significant for screening DEGs.

### Gene ontology (GO) and kyoto encyclopedia of genes and genomes (KEGG) enrichment analyses

R packages ‘clusterProfiler’,‘ggplot2’,‘enrichplot’ and ‘org. Hs.eg.db’ were used to perform GO [26] and KEGG [27] pathway analyses of 93 DEGs. P- and Q-value of <0.05 were considered significant enrichment.

### PPI network construction and univariate Cox regression analysis

The PPI network among the significantly enriched DEGs was constructed using the Search Tool for the Retrieval of Interacting Genes (STRING) [28] online database (https://string-db.org/) and the Cytoscape [29] (version 3.7.2) platform was then utilized to visualize the interactive network among DEGs with a confidence level > 0.95 were used for building network. The top 30 DEGs with the largest number of nodes were filtered by interactions between genes.

Univariate Cox proportional hazards regression was used to further screen for meaningful DEGs with prognostic value, and forest plots were generated using R software package ‘survival’ (FDR<0.05).

Among the first 30 core DEGs of the PPI network with predictive value based on the univariate Cox regression analysis, five hub DEGs with strong predictive value were selected.

### Association of HSD11B1 expression with survival and clinicopathological characteristics

The R packages ‘survival’ (https://CRAN.R-project.org/package=survival, version = 3.1–8) was applied for Kaplan–Meier survival analysis. In addition, Clinical parameters, including age, gender, pathological stage, grade, T/M/N classification, were analyzed by R software and Wilcoxon rank-sum test. P < 0.05 was statistically significant.

### Gene set enrichment analysis (GSEA)

GSEA [30] (version 4.0.2) of the HSD11B1 high expression group was performed in the C2 KEGG gene sets (c2.cp.KEGG.v7.4.symbols.gmt) and C7 immunological gene sets (c7.all.v7.4.symbols.gmt) of the Molecular Signatures Database (MSigDB) [31] to identify the enriched pathways. Each enrichment analysis carried out one thousand times gene set permutations. Pathways with the false discovery rate (FDR) <0.05 and NOM P < 0.05 were considered to be significantly enriched.

### Cell culture

ccRCC cell lines (HK-2, 786-O) was obtained from the Department of Nephrology, the Affiliated Hospital of Qingdao University. HK-2 and 786-O cell lines were cultured in DMEM-F12 and RPMI 1640 with 10% FBS (Gibco) and 1% streptomycin-penicillin at 37°C.

### Western blotting

RIPA (Meilunbio, MA0151) buffer supplemented with protease inhibitors and phosphatase inhibitors was used to extract total protein from cells. The protein samples were electrophoresed on SDS-PAGE gels and transferred to PVDF membranes before being blocked with 5% skimmed milk for 1 h. The membrane was incubated with primary antibodys, including HSD11B1 (1:1500 dilution; Abbkine) and β-actin (1:50,000 dilution; Abcam) at 4°C overnight. Secondary goat anti-mouse IgG-HRP (1:5000 dilution; Abcam) antibody was incubated for 1 hour at room temperature. The proteins were visualized using ECL detection reagents (Meilunbio, MA0186) and quantitatively analyzed by Image J. Western blotting was carried out according to the experimental process of our laboratory [32].

### RT‑qPCR

Total RNA was extracted from cell lines (HK-2, 786-O) using an RNA isolation kit (TIANGEN) following the manufacturer’s protocol. The primers of HSD11B1 were: R: 5ʹ- TGAGAATGAGCATGTCTAGTCC −3ʹ, F:5ʹ- AGCGAGGTCAAAAGAAACTCTA −3ʹ. The expression of HSD11B1 was calculated using the 2− ΔΔCq method [33].

### Immunostaining analysis

Immunohistochemistry staining was performed on tissues. Renal clear cell carcinoma and adjacent para-carcinoma tissues (N = 10) were obtained from the Department of Pathology, Affiliated Hospital of Medical College Qingdao University, Qingdao, China. Immunochemical staining was performed according to our previous experiment procedure [32]. Sections were incubated with antibodies against HSD11B1 (1:200 dilution; Abbkine) and secondary antibody (1:200 dilution; Absin).

### Analysis of TIICs

CIBERSORT [34] analysis tool is a deconvolution algorithm, using R software to run CIBERSORT algorithm. The CIBERSORT analytical tool can accurately estimate the proportion of TIICs from the expression profiles of complex samples. The algorithm calculates the proportion of 22 kinds of TIICs for each ccRCC sample based on LM22 signatures and 1000 matrix permutations. The proportion of immune cells is presented as a bar graph. P < 0.05 can be further correlation analysis.

The Wilcoxon rank-sum test and Spearman’s correlation coefficient were applied to analyze differential and immune cell correlation analyses by using R packages ‘Pheatmap’, ‘Corrplot’ and ‘Vioplot’ to evaluate the association between TIICs and HSD11B1 expression. P < 0.05 was considered statistically significant.

### Statistical analysis

Statistical analysis was performed using GraphPad Prism 5. The unpaired t-test (two-tailed) with confidence intervals of 95% was used to detect the differences in HSD11B1 expression between two independent groups and P < 0 .05 were considered statistically significant.

## Results

In the present research, we aim to establish a predictive model to identify the key genes affecting the prognosis of ccRCC, so as to provide a theoretical basis for predicting the prognosis of ccRCC patients and further seeking new treatment options. In this study, we first downloaded 611 ccRCC gene-expressed data and 537 related clinical information from the Cancer Genome Atlas (TCGA) database. Then we screened HSD11B1 as a possible prognostic biomarker of ccRCC. In addition, we performed association analysis between HSD11B1 and immune cells to evaluate its correlation with tumor microenvironment. Finally, the bioinformatics results were verified by in vitro experiments. We determined that HSD11B1 may be a novel biomarker for the diagnosis and prognosis of ccRCC. This finding is expected to benefit ccRCC patients.

### Correlation analysis between immune/stromal scores and patient survival and clinicopathological characteristics

**Figure 1.**
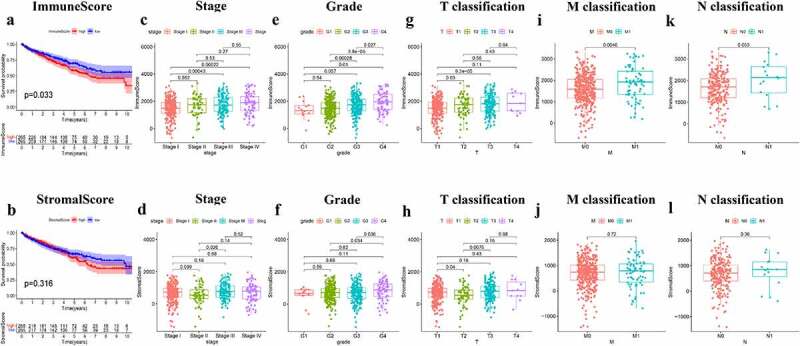
Immune/StromalScores were associated with survival and clinicopathological characteristics. (a-b) Kaplan-Meier curve and survival analysis of ccRCC patients which grouped by the Immune/Stromal Scores was performed. The scores represent the proportion of cells in TME. (c-l) Kruskal-Wallis rank sum test was used to analyze the correlation between immune/stromal components with clinicopathological characteristics (Stage, Grade, T classification, M classification, N classification)

To assess the association between survival rate and immune/stromal scores, 611 ccRCC samples (539 tumor and 72 normal cases) were divided according to scores, and Overall survival analysis was performed. Kaplan-Meier survival curve showed that the proportion of immune cells was notably associated with patients survival (P = 0.033; Figure 1(a)). Although patients with low stromal score had a higher median Overall survival rate than those with higher stromal score, no significant correlation was observed between stromal score and survival (P = 0.316; Figure 1(b)). The aforementioned findings suggested that the proportion of immune cells was closely related to the Overall survival in ccRCC patients.

Furthermore, the paired clinicopathological characteristics of ccRCC patients were collected from TCGA database and their association with the proportion of immune and stromal cells was determined. The analysis revealed that the immune score was closely associated with several clinicopathological characteristics, including pathological stage (stage III vs. I, P = 0.00043; stage IV vs. I, P = 0.00022; Figure 1(c)), grade (G4 vs. G1, P = 0.01; G3 vs. G2, P = 0.00028; G4 vs. G2, P = 3.8E−06; G4 vs. G3, P = 0.027; Figure 1(e)), T classification (T2 vs. T1, P = 0.03; T3 vs. T1, P = 9.2E−05; Figure 1(g)), and M classification (P = 0.0046; Figure 1(i)). However, stromal score was only associated with pathological stage (stage III vs. II, P = 0.026; Figure 1(d)), grade (G4 vs. G2, P = 0.034; G4 vs. G3, P = 0.036; figure 1(f)), and T classification of the tumor-node-metastasis staging system (T2 vs. T1, P = 0.04; T3 vs. T2, P = 0.0075; Figure 1(h)). Overall, the results demonstrated that the proportion of immune/stromal cells was closely related to factors promoting ccRCC progression, such as immune cell infiltration, metastasis and prognosis.

### Correlation between ccRCC gene expression profiles and immune/stromal scores

**Figure 2.**
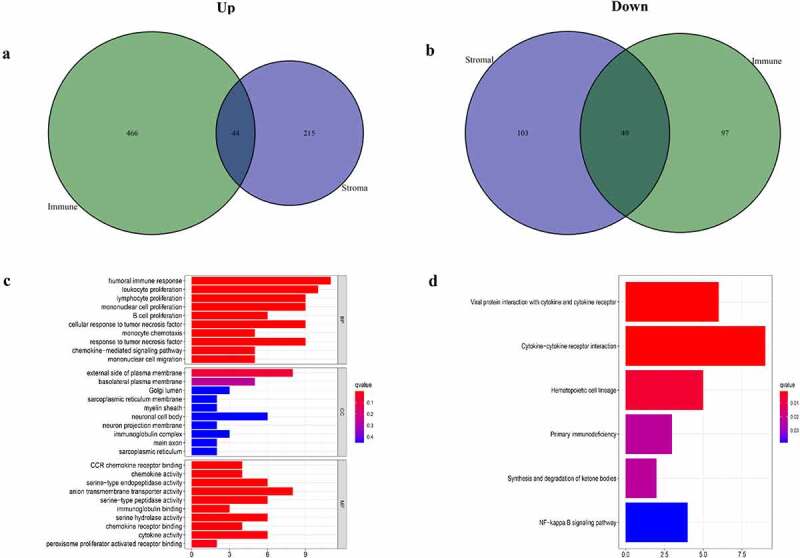
Analyses of gene expression profiles of Immune/StromalScores. (a, b) Venn plot of the number of common upregulated or downregulated DEGs, FDR<0.05, |logFC|>1 were used as the significance threshold. (c, d) GO and KEGG enrichment analyses were used to select DEGs, terms with P < 0.05

ccRCC samples were divided into two groups based on their respective median immune/stromal scores. A total of 656 DEGs were selected according to scores (high vs. low scores). Among them, 510 upregulated genes and 146 downregulated genes were obtained from ImmuneScore (Supplementary Figure 1(a) and Figure 2(a)). Similarly, among the 411 DEGs from StromalScore, 259 and 152 DEGs were up- and downregulated, respectively (Supplementary Figure S1b and Figure 2(b)). Venn plot were used to determine the intersection between the two types of cells. Therefore, 93 common DEGs were overlapping genes in immune and stromal groups, including 44 upregulated and 49 downregulated genes. The aforementioned findings indicated that DEGs may determine the TME status. Subsequently, the top 10 DEGs, significantly enriched in biological processes (BP), cellular component (CC) and molecular function (MF) were selected from GO enrichment analysis (Figure 2(c)). The functional clusters of these DEGs corresponded to immune-related GO terms, such as ‘humoral immune response’ and ‘B-cell proliferation’. KEGG analysis revealed that DEGs were also significantly enriched in immune response-related signaling pathways, including ‘cytokine-cytokine receptor interaction’, ‘primary immunodeficiency’ and ‘cytokine activity’ (Figure 2(d)). Overall, the aforementioned results indicated that DEGs obtained by the intersection of immune/stromal scores were correlated with immune response.

### Identification of DEGs by PPI networks and univariate Cox regression analysis

**Figure 3.**
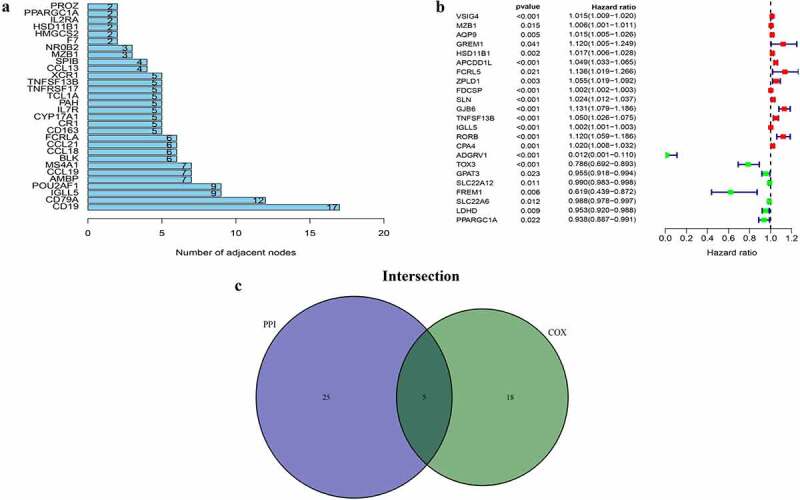
PPI network and univariate cox regression. (a) The first 30 genes sequenced by adjacent nodes numbers in PPI network. (b) The survival of ccRCC patients were performed by univariate cox, with P < 0.05 as a significant difference criterion. (c) Venn plot showed 5 genes that come from the intersection of (A) and (B)

Subsequently, two methods were used to screen for DEGs. Therefore, to study interactions among DEGs, we constructed a PPI network using STRING and Cytoscape software. The top 30 DEGs with the largest number of adjacent nodes were selected for further analysis (Figure 3(a)). Furthermore, univariate Cox regression analysis was used to identify the DEGs significantly associated with prognosis in ccRCC patients (Figure 3(b)). The results of both methods were combined into a Venn plots, and five overlapping genes, namely HSD11B1, TNFSF13B, MZB1, IGLL5 and PPARGC1A, were obtained (Figure 3(c)). The details of the five overlapping genes are presented in Table 2.

**Table 2.** List of 5 kinds of DEGs

Gene name	Gene ID	Description	Location	Expression	Degree score	Log‐rank test of P value*
PPARGC1A	10891	PPARG coactivator 1 alpha	Chr4p15.2	Up-regulated	2	0.022
HSD11B1	3290	hydroxysteroid 11‐beta dehydrogenase 1	Chr1q32.2	Up-regulated	2	0.002
MZB1	51,237	marginal zone B and B1 cell specific protein	Chr5q31.2	Up-regulated	3	0.015
TNFSF13B	10673	TNF superfamily member 13b	Chr13q33.3	Up-regulated	5	&lt;0.001
IGLL5	100,423,062	immunoglobulin lambda‐like polypeptide 5	Chr22q11.22	Up-regulated	9	&lt;0.001

*The P-value showing statistical significance was marked with bold type.

Abbreviation: DEGs, differentially expressed genes.

### Association between HSD11B1 expression levels with survival and clinicopathological characteristics

**Figure 4.**
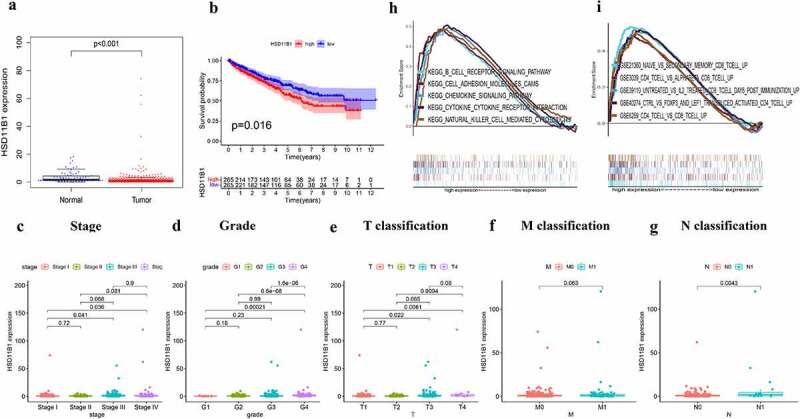
Correlation analysis was applied between HSD11B1 and clinical factors. (a) Wilcoxon rank sum test was used to analyze the expression levels of HSD11B1 in normal and tumor groups with p < 0.05 as the cutoff. (b) Kaplan–Meier analysis of HSD11B1. The survival rate of ccRCC samples decreased over time and the group with low HSD11B1 expression had better survival. (c–g) Wilcoxon rank sum indicated that HSD11B1 expression was correlated with clinicopathological characteristics. (h) GSEA of HSD11B1 high expression group in C2 KEGG gene sets. Unique colored lines represent unique signal pathways of gene set enrichment. The curve above the abscissa indicates the enrichment pathways, and the transverse line below shows the number of genes enriched in each pathway. NOM P < 0.05 was considered to indicate a statistically significant difference. (i) GSEA of HSD11B1 high expression group in C7 immune gene sets

The HSD11B1 gene encodes the type 1 isoform of 11-β-hydroxysteroid dehydrogenase, which converts glucocorticoids into the active form, which plays an important role in the regulation of metabolic syndrome and immune response. This finding suggested that HSD11B1 may play a key role in TME and be a key factor in ccRCC. Therefore, the present study indicated that HSD11B1 potential be a prognostic marker in ccRCC. Herein, ccRCC samples were divided into two groups using the median HSD11B1 expression level as cutoff values. Wilcoxon rank-sum test showed that HSD11B1 expression in tumor group was significantly higher than that in normal group (Figure 4(a)). Furthermore, increased HSD11B1 expression was positively correlation with unfavorable prognosis (Figure 4(b)). In addition, HSD11B1 expression was closely related to several clinicopathological characteristics with ccRCC patients. Therefore, pathological stage (III vs. I, P = 0.041; IV vs. I, P = 0.036), grade (G4 vs. G1, P *= *0.00021; G4 vs. G2, P = 6.6E−08; G4 vs. G3, P = 1.6E−06), T classification (T3 vs. T1, P = 0.022, T4 vs. T1, P = 0.0061, T4 vs. T2, P = 0.0094) and N classification (P = 0.0043) were strongly correlated with HSD11B1 expression (Figure 4(c-g)). Overall, the aforementioned results suggested that HSD11B1 expression levels were negatively related to the prognosis in ccRCC patients.

### GSEA of HSD11B1

As shown in Figure 4(h-i), GSEA was used to evaluate the correlation between HSD11B1 expression with immune response. The bioinformatics analysis demonstrated that several immune-related signaling pathways, including ‘B-cell receptor signaling pathway’, ‘cytokine-cytokine receptor interaction’ and ‘chemokine signaling pathway’ were primarily enriched in C2 KEGG gene sets in HSD11B1 high expression group (Figure 4(h)). In addition, the C7 immunological gene sets (Figure 4(i)) were enriched in the high expression group of HSD11B1, including ‘CD4 T cell vs. CD8 T cell up’, ‘CD4 T cell vs. alphabeta CD8 T cell up’, ‘naive vs. secondary memory CD8 T cell up’. The result showed that HSD11B1 is not only associated with immune-related activities, but may also be a potential biomarker of the TME status.

### Positive expression of HSD11B1 in 786-O cell lines and ccRCC clinical specimens

**Figure 5.**
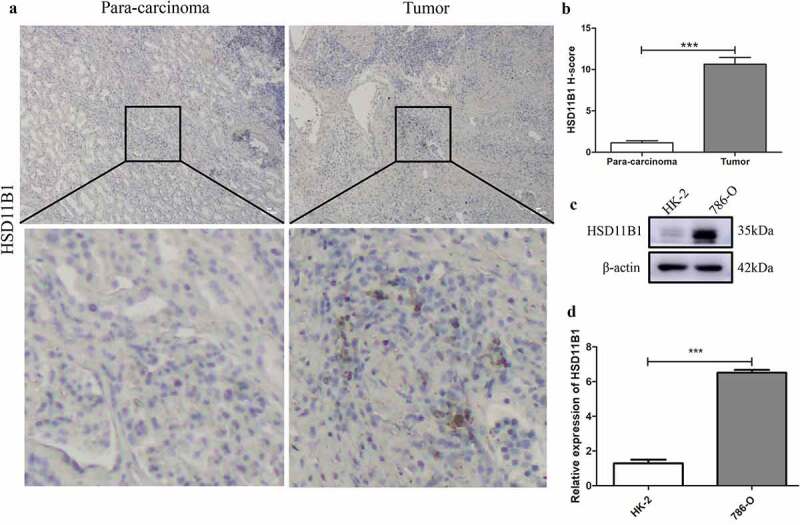
HSD11B1 was significantly abundance in ccRCC cell lines and tissues (a,b) Immunohistochemistry images and analysis of ccRCC clinical specimens and para-carcinoma specimens. (c) Western blot showed that HSD11B1 was overexpressed in 786-O cell lines. (d) RT‑qPCR analysis of high expression of HSD11B1 in 786-O cell line

The high expression of HSD11B1 in cell lines was verified in protein level compared to controls, which is consistent with our bioinformatic analyses (Figure 5(c)). Furthermore, HSD11B1 expression in clinical tissues (Figure 5(a-b)) were observed by immunochemical staining, which showed that significantly higher HSD11B1 expression in ccRCC clinical tissues than that in para-carcinoma specimens. These results confirmed that the abundance of HSD11B1 increased significantly in ccRCC. RT‑qPCR (P&lt;0.0001) showed that HSD11B1 was up-regulated in ccRCC (Figure 5(d)). This result is consistent with the result in Figure 4.

### Association between the expression of HSD11B1 and the proportion of TIICs

**Figure 6.**
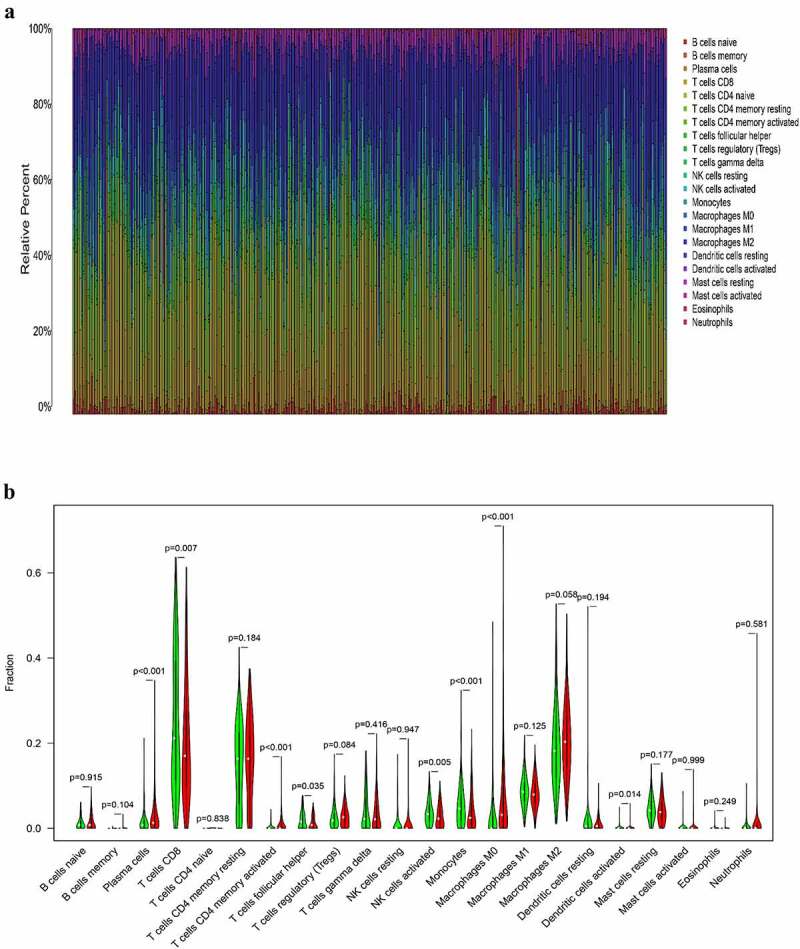
Correlation analysis between TIIC proportion and HSD11B1 expression in ccRCC samples (a) Proportions of 22 types of TIIC in each sample were clearly showed in histogram, sample ID on the horizontal axis and immune cells counts in the sample on the vertical. (b) The violin plot showed the correlation analysis between the infltration level of immune cells and low (green) and high (red) HSD11B1 expression groups relative to the median of HSD11B1 expression. Wilcoxon rank sum test was commonly believed to be significantly

**Figure 7.**
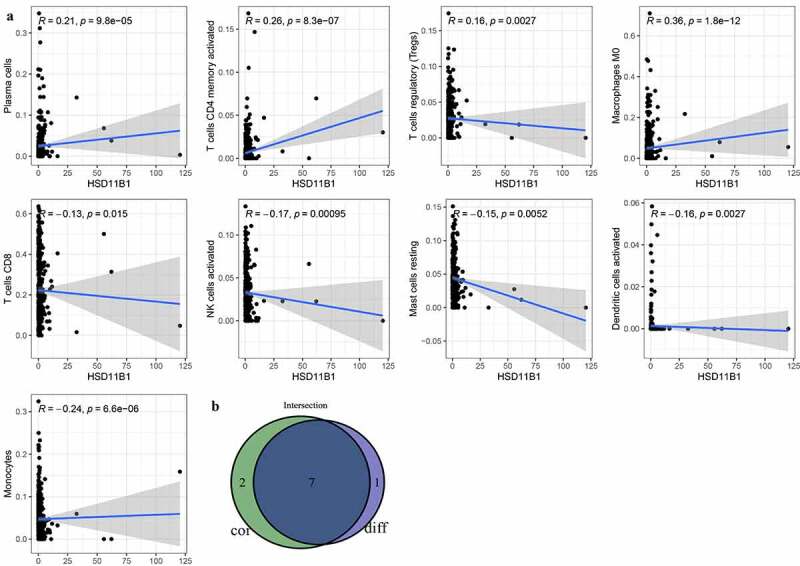
Tumor microenvironment exploration of HSD11B1 (a) Correlation analysis between HSD11B1 expression and TIIC proportion was calculated by scatter plot. The correlation coefficient R > 0 in the scatter plot indicated a positive correlation with HSD11B1 and tumor purity and immune cells. There exist a linear correlation equation between the infltration level of immune cells and HSD11B1 expression. Correlations test was evaluated using Pearson coefficient. (b) The Venn plot showed 7 types of TIIC associated with HSD11B1 expression identified by the intersection of the differential immune cells in the violin plot and the scatter plot

To further elucidate the association between HSD11B1 expression and the TME status in ccRCC, the proportion of TIICs in ccRCC was analyzed using the CIBERSORT algorithm (Figure 6(a)). The violin plot (Figure 6(b)) which illustrating the profile of 22 immune cell subpopulations showing that there were statistically significant differences in Plasma cells (P&lt;0.001), T cells CD8 (P = 0.007), T cells CD4 memory activated (P&lt;0.001), T cells follicular helper (P = 0.035), NK cells activated (P = 0.005), Monocytes (P&lt;0.001), Macrophages M0 (P&lt;0.001), Dendritic cells activated (P = 0.014). In addition, we found there were statistically significant differences in Plasma cells (R = 0.21, P = 9.8E-05), T cells CD8 (R = ‐0.13, P = 0.015), T cells CD4 memory activated (R = 0.26, P = 8.3E-07), T cells regulatory (Tregs) (R = 0.16, P = 0.0027), NK cells activated (R = ‐0.17, P = 0.00095), Monocytes (R = ‐0.24, P = 6.6E-06), Macrophages M0 (R = 0.36, P = 1.8E-12), Dendritic cells activated (R = ‐0.16, P = 0.0027), Mast cells resting (R = ‐0.15, P = 0.0052) through Spearman’s correlation coefficient (Figure 7(a)). Subsequently, the results of the differential analysis and those from the immune cell association analysis were intersected (Figure 7(b), Table 3).

**Table 3.** TIICs co-determined by difference test and correlation test

TIICs	Difference test (P-Value)	Correlation test(P-Value)
Plasma cells	0.000259169699189331	9.7681078980995E-05
T cells CD8	0.006788819191731	0.0148198239813821
T cells CD4 memory activated	0.00010274900899535	8.30626484098104E-07
NK cells activated	0.00455656929361048	0.00094723708787107
Monocytes	0.000454239467850045	6.59316505377974E-06
Macrophages M0	4.43187411856744e-09	1.75555276773066E-12
Dendritic cells activated	0.01368813484952	0.00271480919176661

Abbreviation: TIIC, Tumor-infiltrating immune cell.

## Discussion

In the present study, ccRCC samples from TCGA database were used to elucidate the genes associated with prognosis. Bioinformatics analysis results indicated that HSD11B1 could be a novel prognostic biomarker of ccRCC.

TME serves an important part in tumor development and progression. Nowadays, the research into potential prognostic targets for advanced renal cancer is growing rapidly. Compared with traditional surgical resection, patients undergoing targete.d therapy have a significantly longer survival time [7,35,36]. Identifying potential prognostic biomarkers is of great importance for the diagnosis, treatment and prognosis of cancer targeted immunotherapy. Transcriptome analysis of ccRCC in TCGA database indicated a close association between ImmuneScore with survival of ccRCC patients. Furthermore, it was significantly correlated with multiple clinicopathologic characteristics of TCGA-ccRCC samples, including grade, pathological stage, T classification, and M classification. It further demonstrated that the components of the immune system within TME could serve a vital role in the development and prognosis of ccRCC. GO analysis confirmed a significant enrichment of DEGs in immune-related terms. Emerging evidence has suggested that immune responses promote the development of several types of cancer [37–39]. Herein, KEGG enrichment analysis showed that DEGs were closely linked to immune-related activities. These findings indicated that DEGs may have a significant impact on regulating TME and influencing the prognosis of ccRCC. Since The HSD11B1 gene encodes the type 1 isoform of 11-β-hydroxysteroid dehydrogenase, which converts glucocorticoids into the active form, which plays an important role in the regulation of metabolic syndrome and immune response [21]. Further analyses were carried out on HSD11B1 expression profile. These studies revealed that HSD11B1 was upregulated in ccRCC samples and can be considered as prognostic indicator of ccRCC, as predicted by univariate Cox regression analysis. HSD11B1 elevated expression was significantly related to poor survival rate and several clinicopathological characteristics. The results obtained by western blotting, RT‑qPCR and immunohistochemistry were consistent with the results of this study. Overall, these findings demonstrated convincingly that HSD11B1 is a potential valuable prognostic marker.

HSD11B1 is a member of the short-chain dehydrogenase/ reductase (SDR) superfamily, which is the only enzyme in the body that converts inactive cortisone into active glucocorticoid hormones [40]. The enzyme is abundant in liver and can be induced to express in immune cells. Previous studies have shown that glucocorticoids encoded by HSD11B1 contribute to the regulation of cell proliferation and differentiation, and recognized the important role HSD11B1 play in promoting the development and progression of colon tumors and adenocarcinoma cells [22,23,41,42]. Proliferative phenotypes induced by high expression of HSD11B1 are associated with poor cancer prognosis [23]. Herein, increased HSD11B1 expression was interconnected with poor survival of ccRCC patients, supporting a potential association between HSD11B1 expression and tumor progression and clinical outcome. These findings indicated that HSD11B1 could act as oncogene in ccRCC, which was consistent with the tumor-promoting effect of HSD11B1 on other types of cancer. GSEA showed that HSD11B1 expression was associated with several immune-related signaling pathways, suggesting that the immune responses may be activated during the progression of ccRCC. In addition, the analysis revealed that the up-regulation of chemokine signaling pathway also promoted the development of CCRCC. Previous studies have shown that chemokines, such as CXCL13 and CXCR5, are crucial for the progression and poor prognosis of ccRCC [43,44].

The results also demonstrated that seven kinds of TIICs were significantly associated with the TME status in ccRCC. In the present study, the proportion of Macrophages M0 was positively associated with HSD11B1 expression. Compared with the low HSD11B1 expression group, the number of Macrophages M0 was obvious abundant in HSD11B1 high expression group. Clinical trials indicated that Macrophages M0, as inhibitors of the antitumor immune responses, were related to poor prognosis in sizable proportion of all cancers, such as lung [45,46], prostate [47], colorectal [48], breast [49,50], hepatocellular [51] and head and neck [52] cancer. Numerous studies have verified that macrophages, as an important member of myeloid origin cells in TME, can participate in the occurrence, development and immunosuppression of tumors [53]. Another immunosuppressive cell type in cancer, Plasma cells, may significantly affect the survival of patients with ccRCC. Plasma cells are known to be products of B-cell differentiation that play a key role in humoral immune system by producing and secreting antibodies [54]. More and more studies have shown that plasma cells have become a significantly survival biomarkers for a variety of solid tumors [55,56]. Recent studies have also confirmed that the results of the present study, demonstrating that increased numbers of Plasma cells were closely associated with poor survival. In the majority of solid tumors, CD8 T-cell infiltration is often associated with good prognosis [57]. Consistently, the results of this study showed a negative correlation between CD8 T cell infiltration and HSD11B1 expression. Our results support B cells provided the robust anti-tumor immunity by served as antigen-presenting cells [58,59]. Other studies have reported that immune cells, for instance, NK cells activated and Dendritic cells activated are involved in tumor immune surveillance and exert antitumor effects. A study showed that NK cells could mediate cytolysis to induce apoptosis of target cells [60]. In addition, NK cells can enhance the antitumor activity via secreting pro-inflammatory chemokines and cytokines [61]. A recent study demonstrated that NK cells, as immune effector cells, were potentially involved in the treatment of kidney cancer via enhancing chimeric antigen receptor (CAR) modification [62]. As mentioned above, active Dendritic cells can activate tumor immunity and aggregate immune effector cells to the tumor site [63]. Therefore, the negative association of the proportion of NK cells activated, Dendritic cells activated with the expression levels of HSD11B1 in ccRCC patients indicated that HSD11B1 potential play a pivotal role in tumorigenesis and development.

## Conclusion

In conclusion, the results of the present study supported that the interaction between ccRCC and TME affected cancer progression, thereby affecting the overall prognosis of patients with ccRCC. The analysis of TCGA-ccRCC patient samples and in-vitro experimental verification revealed that HSD11B1 as a potential prognostic indicator of ccRCC. Finally, further studies on HSD11B1 could provide a foundation for understanding the complex association between TME and the prognosis of patients with ccRCC.

## Supplementary Material

Supplemental Material

## Data Availability

The datasets generated and analyzed during this study are available in the TCGA database (https://portal.gdc.cancer.gov).

## Highlights

- The prognosis of ccRCC was correlated with immune regulation of TME.
- The expression level of HSD11B1 was correlated with the prognosis of ccRCC patients.
- HSD11B1 can be used as a prognostic marker in TME of ccRCC.

## References

[1] Song M. Recent developments in small molecule therapies for renal cell carcinoma. Eur J Med Chem. 2017 Dec 15;142: 383–392. 28844802 10.1016/j.ejmech.2017.08.007

[2] Motzer RJ, Jonasch E, Agarwal N, et al. Kidney cancer, version 2.2017, NCCN clinical practice guidelines in oncology. J Natl Compr Canc Netw. 2017 Jun;15(6):804–834. 28596261 10.6004/jnccn.2017.0100

[3] Siegel RL, Miller KD, Jemal A. Cancer statistics, 2020. CA Cancer J Clin. 2020 Jan;70(1):7–30. 31912902 10.3322/caac.21590

[4] Frew IJ, Moch H. A clearer view of the molecular complexity of clear cell renal cell carcinoma. Annu Rev Pathol. 2015;10:263–289. 25387056 10.1146/annurev-pathol-012414-040306

[5] Jonasch E, Walker CL, Rathmell WK. Clear cell renal cell carcinoma ontogeny and mechanisms of lethality. Nat Rev Nephrol. 2021 Apr;17(4):245–261. 33144689 10.1038/s41581-020-00359-2 PMC8172121

[6] Procházková K, Vodička J, Fichtl J, et al. Outcomes for patients after resection of pulmonary metastases from clear cell renal cell carcinoma: 18 years of experience. Urol Int. 2019;103(3):297–302. 31434090 10.1159/000502493

[7] Motzer RJ, Bander NH, Nanus DM. Renal-cell carcinoma. N Engl J Med. 1996 Sep 19;335(12):865–875. 8778606 10.1056/NEJM199609193351207

[8] Ricketts CJ, De Cubas AA, Fan H, et al. The cancer genome atlas comprehensive molecular characterization of renal cell carcinoma. Cell Rep. 2018 Apr 3;23(1):313–326.e5. 29617669 10.1016/j.celrep.2018.03.075 PMC6075733

[9] Adashek JJ, Salgia MM, Posadas EM, et al. Role of biomarkers in prediction of response to therapeutics in metastatic renal-cell carcinoma. Clin Genitourin Cancer. 2019 Jun;17(3):e454–e460. 30733185 10.1016/j.clgc.2019.01.004

[10] Lu Q, Zhang Y, Chen X, et al. Prognostic significance and immune infiltration of microenvironment-related signatures in pancreatic cancer. Medicine (Baltimore). 2021 Mar 26;100(12):e24957. 33761652 10.1097/MD.0000000000024957 PMC9282111

[11] Chevrier S, Levine JH, Zanotelli VRT, et al. An immune atlas of clear cell renal cell carcinoma. Cell. 2017 May 4;169(4):736–749.e18. 28475899 10.1016/j.cell.2017.04.016 PMC5422211

[12] Hanahan D, Weinberg RA. Hallmarks of cancer: the next generation. Cell. 2011 Mar 4;144(5):646–674. 21376230 10.1016/j.cell.2011.02.013

[13] Quail DF, Joyce JA. Microenvironmental regulation of tumor progression and metastasis. Nat Med. 2013 Nov;19(11):1423–1437. 24202395 10.1038/nm.3394 PMC3954707

[14] Garcia-Gomez A, Rodríguez-Ubreva J, Ballestar E. Epigenetic interplay between immune, stromal and cancer cells in the tumor microenvironment. Clin Immunol. 2018 Nov;196:64–71. 29501540 10.1016/j.clim.2018.02.013

[15] Şenbabaoğlu Y, Gejman RS, Winer AG, et al. Tumor immune microenvironment characterization in clear cell renal cell carcinoma identifies prognostic and immunotherapeutically relevant messenger RNA signatures. Genome Biol. 2016 Nov 17;17(1):231. 27855702 10.1186/s13059-016-1092-z PMC5114739

[16] Wolf MM, Kimryn Rathmell W, Beckermann KE. Modeling clear cell renal cell carcinoma and therapeutic implications. Oncogene. 2020 Apr;39(17):3413–3426. 32123314 10.1038/s41388-020-1234-3 PMC7194123

[17] Vuong L, Kotecha RR, Voss MH, et al. Tumor microenvironment dynamics in clear-cell renal cell carcinoma. Cancer Discov. 2019 Oct;9(10):1349–1357. 31527133 10.1158/2159-8290.CD-19-0499 PMC6774890

[18] Gajewski TF, Schreiber H, Fu YX. Innate and adaptive immune cells in the tumor microenvironment. Nat Immunol. 2013 Oct;14(10):1014–1022. 24048123 10.1038/ni.2703 PMC4118725

[19] Yoshihara K, Shahmoradgoli M, Martínez E, et al. Inferring tumour purity and stromal and immune cell admixture from expression data. Nat Commun. 2013;4:2612. 24113773 10.1038/ncomms3612 PMC3826632

[20] Chen B, Khodadoust MS, Liu CL, et al. Profiling tumor infiltrating immune cells with CIBERSORT. Methods Mol Biol. 2018;1711:243–259. 29344893 10.1007/978-1-4939-7493-1_12 PMC5895181

[21] Feldman K, Likó I, Nagy Z, et al. [Importance of the 11β-hydroxysteroid dehydrogenase enzyme in clinical disorders]. Orv Hetil. 2013 Feb 24;154(8):283–293.A 11-β-hidroxi-szteroid-dehidrogenáz enzim jelentősége klinikai kórképekben. 23419529 10.1556/OH.2013.29558

[22] Wang J, Gao Y, Wang L, et al. A variant (rs932335) in the HSD11B1 gene is associated with colorectal cancer in a Chinese population. Eur J Cancer Prev. 2013 Nov;22(6):523–528. 24061267 10.1097/CEJ.0b013e3283656346

[23] Li CF, Liu TT, Wang JC, et al. Hydroxysteroid 11-beta dehydrogenase 1 overexpression with copy-number gain and missense mutations in primary gastrointestinal stromal tumors. J Clin Med. 2018;7:11. 10.3390/jcm7110408 PMC6262574 30388854

[24] Tomczak K, Czerwińska P, Wiznerowicz M. The Cancer Genome Atlas (TCGA): an immeasurable source of knowledge. Contemp Oncol (Pozn). 2015;19(1a):A68–77. 25691825 10.5114/wo.2014.47136 PMC4322527

[25] Ritchie ME, Phipson B, Wu D, et al. limma powers differential expression analyses for RNA-sequencing and microarray studies. Nucleic Acids Res. 2015 Apr 20;43(7):e47. 25605792 10.1093/nar/gkv007 PMC4402510

[26] Thomas PD. The gene ontology and the meaning of biological function. Methods Mol Biol. 2017;1446:15–24. 27812932 10.1007/978-1-4939-3743-1_2 PMC6438694

[27] Kanehisa M, Furumichi M, Tanabe M, et al. KEGG: new perspectives on genomes, pathways, diseases and drugs. Nucleic Acids Res. 2017 Jan 4;45(D1):D353–d361. 27899662 10.1093/nar/gkw1092 PMC5210567

[28] Szklarczyk D, Gable AL, Lyon D, et al. STRING v11: protein-protein association networks with increased coverage, supporting functional discovery in genome-wide experimental datasets. Nucleic Acids Res. 2019 Jan 8;47(D1):D607–d613. 30476243 10.1093/nar/gky1131 PMC6323986

[29] Shannon P, Markiel A, Ozier O, et al. Cytoscape: a software environment for integrated models of biomolecular interaction networks. Genome Res. 2003 Nov;13(11):2498–2504. 14597658 10.1101/gr.1239303 PMC403769

[30] Subramanian A, Tamayo P, Mootha VK, et al. Gene set enrichment analysis: a knowledge-based approach for interpreting genome-wide expression profiles. Proc Natl Acad Sci U S A. 2005 Oct 25;102(43):15545–15550. 16199517 10.1073/pnas.0506580102 PMC1239896

[31] Liberzon A, Subramanian A, Pinchback R, et al. Molecular signatures database (MSigDB) 3.0. Bioinformatics. 2011 Jun 15;27(12):1739–1740. 21546393 10.1093/bioinformatics/btr260 PMC3106198

[32] Mu H, Wang Z, Zhang X, et al. HCMV-encoded IE2 induces anxiety-depression and cognitive impairment in UL122 genetically-modified mice. Int J Clin Exp Pathol. 2019;12(11):4087–4095. 31933804 PMC6949793

[33] Livak KJ, Schmittgen TD. Analysis of relative gene expression data using real-time quantitative PCR and the 2(-Delta Delta C(T)) method. Methods. 2001 Dec;25(4):402–408. 11846609 10.1006/meth.2001.1262

[34] Newman AM, Liu CL, Green MR, et al. Robust enumeration of cell subsets from tissue expression profiles. Nat Methods. 2015 May;12(5):453–457. 25822800 10.1038/nmeth.3337 PMC4739640

[35] Kumar R, Kapoor A. Current management of metastatic renal cell carcinoma: evolving new therapies. Curr Opin Support Palliat Care. 2017 Sep;11(3):231–237. 28590313 10.1097/SPC.0000000000000277

[36] Choueiri TK, Motzer RJ. Systemic therapy for metastatic renal-cell carcinoma. N Engl J Med. 2017 Jan 26;376(4):354–366. 28121507 10.1056/NEJMra1601333

[37] Miranda A, Hamilton PT, Zhang AW, et al. Cancer stemness, intratumoral heterogeneity, and immune response across cancers. Proc Natl Acad Sci U S A. 2019 Apr 30;116(18):9020–9029. 30996127 10.1073/pnas.1818210116 PMC6500180

[38] Hays E, Bonavida B. YY1 regulates cancer cell immune resistance by modulating PD-L1 expression. Drug Resist Updat. 2019 Mar;43:10–28. 31005030 10.1016/j.drup.2019.04.001

[39] Wagner J, Rapsomaniki MA, Chevrier S, et al. A single-cell atlas of the tumor and immune ecosystem of human breast cancer. Cell. 2019 May 16;177(5):1330–1345.e18. 30982598 10.1016/j.cell.2019.03.005 PMC6526772

[40] Chedid MF, do Nascimento FV, de Oliveira FS, et al. Interaction of HSD11B1 and H6PD polymorphisms in subjects with type 2 diabetes are protective factors against obesity: a cross-sectional study. Diabetol Metab Syndr. 2019;11:78. 31558916 10.1186/s13098-019-0474-2 PMC6755690

[41] Modesto JL, Hull A, Angstadt AY, et al. NNK reduction pathway gene polymorphisms and risk of lung cancer. Mol Carcinog. 2015 Jun;54(Suppl 1):E94–e102. 24976539 10.1002/mc.22187 PMC6296469

[42] Hu D, Zhou M, Zhu X. Deciphering immune-associated genes to predict survival in clear cell renal cell cancer. Biomed Res Int. 2019;2019:2506843. 31886185 10.1155/2019/2506843 PMC6925759

[43] Xu T, Ruan H, Song Z, et al. Identification of CXCL13 as a potential biomarker in clear cell renal cell carcinoma via comprehensive bioinformatics analysis. Biomed Pharmacother. 2019 Oct;118:109264. 31390578 10.1016/j.biopha.2019.109264

[44] Zheng Z, Cai Y, Chen H, et al. CXCL13/CXCR5 axis predicts poor prognosis and promotes progression through PI3K/AKT/mTOR pathway in clear cell renal cell carcinoma. Front Oncol. 2018;8:682. 30723697 10.3389/fonc.2018.00682 PMC6349755

[45] Liu X, Wu S, Yang Y, et al. The prognostic landscape of tumor-infiltrating immune cell and immunomodulators in lung cancer. Biomed Pharmacother. 2017 Nov;95:55–61. 28826097 10.1016/j.biopha.2017.08.003

[46] Zhong R, Chen D, Cao S, et al. Immune cell infiltration features and related marker genes in lung cancer based on single-cell RNA-seq. Clin Transl Oncol. 2021 Feb;23(2):405–417. 32656582 10.1007/s12094-020-02435-2

[47] Meng J, Liu Y, Guan S, et al. The establishment of immune infiltration based novel recurrence predicting nomogram in prostate cancer. Cancer Med. 2019 Sep;8(11):5202–5213. 31355524 10.1002/cam4.2433 PMC6718526

[48] Ge P, Wang W, Li L, et al. Profiles of immune cell infiltration and immune-related genes in the tumor microenvironment of colorectal cancer. Biomed Pharmacother. 2019 Oct;118:109228. 31351430 10.1016/j.biopha.2019.109228

[49] Ali HR, Chlon L, Pharoah PD, et al. Patterns of immune infiltration in breast cancer and their clinical implications: a gene-expression-based retrospective study. Plos Med. 2016 Dec;13(12):e1002194. 27959923 10.1371/journal.pmed.1002194 PMC5154505

[50] Bense RD, Sotiriou C, Piccart-Gebhart MJ, et al. Relevance of tumor-infiltrating immune cell composition and functionality for disease outcome in breast cancer. J Natl Cancer Inst. 2017 Jan;109:1. 10.1093/jnci/djw192 PMC6284248 27737921

[51] Zhang Y, Zhang L, Xu Y, et al. Immune-related long noncoding RNA signature for predicting survival and immune checkpoint blockade in hepatocellular carcinoma. J Cell Physiol. 2020 Dec;235(12):9304–9316. 32330311 10.1002/jcp.29730

[52] Song J, Deng Z, Su J, et al. Patterns of immune infiltration in HNC and their clinical implications: a gene expression-based study. Front Oncol. 2019;9:1285. 31867268 10.3389/fonc.2019.01285 PMC6904960

[53] Gabrilovich DI, Nagaraj S. Myeloid-derived suppressor cells as regulators of the immune system. Nat Rev Immunol. 2009 Mar;9(3):162–174. 19197294 10.1038/nri2506 PMC2828349

[54] Radbruch A, Muehlinghaus G, Luger EO, et al. Competence and competition: the challenge of becoming a long-lived plasma cell. Nat Rev Immunol. 2006 Oct;6(10):741–750. 16977339 10.1038/nri1886

[55] Berntsson J, Nodin B, Eberhard J, et al. Prognostic impact of tumour-infiltrating B cells and plasma cells in colorectal cancer. Int J Cancer. 2016 Sep 1;139(5):1129–1139. 27074317 10.1002/ijc.30138

[56] Fristedt R, Borg D, Hedner C, et al. Prognostic impact of tumour-associated B cells and plasma cells in oesophageal and gastric adenocarcinoma. J Gastrointest Oncol. 2016 Dec;7(6):848–859. 28078109 10.21037/jgo.2016.11.07 PMC5177573

[57] Vano YA, Petitprez F, Giraldo NA, et al. Immune-based identification of cancer patients at high risk of progression. Curr Opin Immunol. 2018 Apr;51:97–102. 29554496 10.1016/j.coi.2018.03.005

[58] Wennhold K, Shimabukuro-Vornhagen A, von Bergwelt-Baildon M. B cell-based cancer immunotherapy. Transfus Med Hemother. 2019 Feb;46(1):36–46. 31244580 10.1159/000496166 PMC6558332

[59] Bi KW, Wei XG, Qin XX, et al. BTK has potential to be a prognostic factor for lung adenocarcinoma and an indicator for tumor microenvironment remodeling: a study based on TCGA data mining. Front Oncol. 2020;10:424. 32351880 10.3389/fonc.2020.00424 PMC7175916

[60] Voskoboinik I, Smyth MJ, Trapani JA. Perforin-mediated target-cell death and immune homeostasis. Nat Rev Immunol. 2006 Dec;6(12):940–952. 17124515 10.1038/nri1983

[61] Guillerey C, Huntington ND, Smyth MJ. Targeting natural killer cells in cancer immunotherapy. Nat Immunol. 2016 Aug 19;17(9):1025–1036. 27540992 10.1038/ni.3518

[62] Zhang Q, Tian K, Xu J, et al. Synergistic effects of cabozantinib and EGFR-specific CAR-NK-92 cells in renal cell carcinoma. J Immunol Res. 2017;2017:6915912. 29423418 10.1155/2017/6915912 PMC5750507

[63] Gardner A, Ruffell B. Dendritic cells and cancer immunity. Trends Immunol. 2016 Dec;37(12):855–865. 27793569 10.1016/j.it.2016.09.006 PMC5135568

