# Supplementary material for: Microenvironment-associated gene HSD11B1 may serve as a prognostic biomarker in clear cell renal cell carcinoma: a study based on TCGA, RT‑qPCR, Western blotting, and immunohistochemistry
Source: Bioengineered. 2021 Nov 30;12(2):10891–904. doi: 10.1080/21655979.2021.1994908 (PMC8810109; doi:10.1080/21655979.2021.1994908)
Supplement: Supplemental Material [file KBIE_A_1994908_SM1236.zip › Supplementary Figure 1 caption.docx]

**Supplementary Figure 1** Heatmap for the DEGs between the high/low ImmuneScore (A) and StromalScore (B) through Wilcoxon rank sum test (FDR<0.05, |logFC|>1). The gene name is called the row name of heatmap and the ID of samples not shown in heatmap is the column name.
